# Mutual Lewis Acid–Base Interactions of Cations and Anions in Ionic Liquids

**DOI:** 10.1002/chem.201201978

**Published:** 2012-11-23

**Authors:** Markus Holzweber, Ralf Lungwitz, Denise Doerfler, Stefan Spange, Mihkel Koel, Herbert Hutter, Wolfgang Linert

**Affiliations:** [a] Vienna University of Technology, Institute of Chemical Engineering and AnalyticsGetreidemarkt 9/164.AC, 1060 Vienna (Austria); [b] Chemnitz University of Technology, Institute of ChemistryStrasse der Nationen 62, 09111 Chemnitz (Germany); [c] Vienna University Of Technology, Institute of Applied Synthetic ChemistryGetreidemarkt 9/163-AC, 106 0 Vienna (Austria); [d] Tallinn Technical University, Institute of ChemistryAkadeemia Tee 15, Tallinn 12618 (Estonia)

**Keywords:** acceptor number, donor number, ionic liquids, solvatochromism

## Abstract

Solute properties are known to be strongly influenced by solvent molecules due to solvation. This is due to mutual interaction as both the properties of the solute and of the solvent strongly depend on each other. The present paper is based on the idea that ionic liquids are cations solvated by anions and anions solvated by cations. To show this (in this system strongly pronounced) interaction the long time established donor–acceptor concept for solvents and ions in solution by Viktor Gutmann is extended to ionic liquids. A number of solvent parameters, such as the Kamlet–Abboud–Taft and the Dimroth–Reichardt *E*_T_ scale for ionic liquids neglect this mutual influence, which, however, seems to be in fact necessary to get a proper description of ionic liquid properties. It is shown how strong such parameters vary when the influence of the counter ion is taken into account. Furthermore, acceptor and donor numbers for ionic liquids are presented.

## Introduction

The selection of an appropriate solvent or solvent mixture to perform chemical reactions or to separate mixtures of reactants and products is crucial in many chemical processes.^1^ Also physical properties such as the frequencies and intensities of transitions in IR, UV-visible, fluorescence, NMR and ESR spectroscopies are also known to be affected by solvents. It is commonly stated that these effects reflect the influence of “solvent polarity”.^2^ A common method to classify solvent systems (including ionic liquids as solvents) and therefore choosing an appropriate solvent is their polarity. Polarity is a general term that refers to all interaction forces between molecules, both specific and nonspecific, excluding such interactions leading to definite chemical alterations of the ions or molecules of the solute.^3^ For better understanding of polarity it is important to describe the interactions between the solute and solvent on molecular level. The most common measure of polarity used by chemists is that of dielectric constant ε. Due to the often observed inadequacies of the dielectric approach another one is needed. To solve this problem empirical solvent parameters have been applied. The basic concept of such an approach is that a particular reaction rate, equilibrium or spectral effect is suitable to serve as a model for other reactions.^4^ Several polarity scales^2^ exist, but the donor–acceptor approach developed by Gutmann^5^, ^6^ precedes all other polarity scales and plays a seminal role in solution chemistry.^7^, ^8^ In this approach, the donor number (DN) is a quantitative measure of Lewis basicity and the acceptor number (AN) a quantitative measure of Lewis acidity, respectively. It has been shown, that Kamlet–Abboud–Taft solvent descriptors do not offer a correlation with the Eyring activation parameters.^9^ Recently in a study of salts (Kosover’s salt) in ionic liquids contradictory results arose;^10^ it has been indicated from measurements of absorption maxima that ionic liquids are of moderate polarity whereas absorptivity studies of the same ionic liquids show that they are highly polar. It was stated, that these results will require new models of solvation and polarity. The proposed donor–acceptor approach to ionic liquids takes a moderating effect of the counter ion (mutual influence), as proposed by Welton et al. in 2003,^11^ into account. This is not included in the well-known Kamlet–Abboud–Taft and Dimroth–Reichardt *E*_T_ polarity scales. AN and DN were determined by spectroscopic methods (UV/Vis) using the solvatochromic indicators Fe(phen)_2_(CN)_2_ for AN and Cu(acac)(tmen)^+^ for DN. Solvatochromism^12^ describes the changes in spectra of dissolved species depending of the media. Such effects are used to visualize solvent properties; however, different indicator species may measure different aspects of solvent solute interactions. This can be overcome in some extent by using multi parameter correlations.

The determination of AN and DN for solvents is carried out straightforward by dissolving the appropriate indicator and measuring the shift of the absorption maximum in the respective solvent to a reference solvent (i.e., dichloroethane for AN and nitromethane for DN).^13^ A linear correlation of the energy related absorption maxima (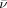 values) of the corresponding indicator in a large number of solvents with the solvent acceptor number AN and the solvent donor number DN, respectively, leads to the following correlations with *r* as correlation factor:^14^–^18^

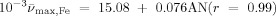
1

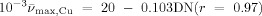
2

The determination of donor and acceptor numbers for ions in non-aqueous solvents is more complex. The ability of anions (cations) to act as an electron pair donor (an electron pair acceptor) influences the donor and acceptor properties of a solvent. The interaction of the cations with the iron indicator depends on both the acceptor and donor properties of the solvent. If the solvent is a stronger Lewis acid than the cation, the solvent interacts solely with the indicator, or the solvent is a Lewis base strong enough to solvate the cations making an interaction between indicator and cation impossible. In these two cases no significant change in the absorption maximum is found as the influence of the dissolved cation is negligible (solvent dependent part). An increase of Lewis acidity and therefore an increase of acceptor properties of the cation leads to a shift of the absorption maximum to lower energies (cation dependent part) (see Figure 5 in ref. 17). This part depends on the acceptor and donor properties of the respective solvent and can be, using nitromethane (NM) as reference solvent (DN=2.7, AN=20.5), expressed as:^17^

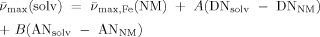
3

A multiple regression analysis of the data yields for *A*=−48.7 and for *B*=−5.62 (*r*=0.96). At the intersection point of the solvent dependent part with the cation dependent part, both the acceptor properties of the solvent and those of the cation are equal and the acceptor number thus obtained is defined as the apparent acceptor number of the cation dissolved in that particular solvent (AN_M,solv_):^17^

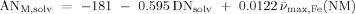
4

The interaction of the anions with the copper indicator also depends on both the acceptor and donor properties of the solvent. If the donor strength of the solvent is higher than that of the anion the solvent coordinates preferably with the copper complex, however, if the acceptor strength of the solvent is too high it will compete with the indicator for the coordination of the anion and no change in the absorption maximum is found (solvent dependent part). If the donor strength of the anion exceeds the donor strength of the solvent a shift of the absorption maximum can be observed (ion dependent part). For strong acceptor solvents such as water and methanol even strong donating anions cannot coordinate to the copper complex as they are preferentially solvated by such solvents (see Figure 3 in ref. 16. The ascending part of the curves depends therefore on both the acceptor and donor number of the respective solvent. The following relationship using dichloroethane (DCE) as reference solvent (DN=0, AN=16.7) can therefore be expressed as:^16^

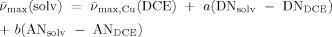
5

A multiple regression analysis of the data yields for *a*=63 and for *b*=91 (*r*=0.97). At the intersection point of the solvent dependent part with the anion dependent part, both the donor properties of the solvent and those of the anion are equal and the donor number thus obtained is defined as the apparent donor number of the anion dissolved in that particular solvent (DN_X,solv_):^16^

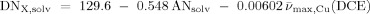
6

The same concept for ions in non-aqueous solutions shall be applied for the determination of donor and acceptor numbers of ionic liquids. As stated above cations and anions influence the donor and acceptor properties of a solvent thus shifting the absorption maximum of a solvatochromic dye. In the case of ionic liquids the assumption is made that cations are solvated by anions and anions solvated by cations. The anions therefore are acting as solvent for the cations and the cations as solvent for the anions. Therefore there is again a mutual influence of the ions on their donating and accepting ability. A system of equations can be established in order to reflect the ionic influence:

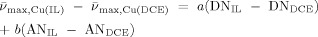
7

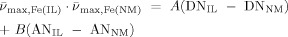
8

## Results and Discussion

By solving the system of Equations (7) and (8), using the previously determined constants (*a*, *b* and *A*, *B*), donor and acceptor numbers for neat ionic liquids can be obtained. Using again dichloroethane and nitromethane as reference solvents the DN and AN are on the same scale as Gutmann’s DN and AN for solvents.^6^ Solving Equations (7) and (8) and inserting parameters, acceptor and donor numbers for neat ionic liquids can easily be determined:

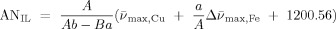
9

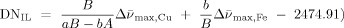
10

**Figure 1:**
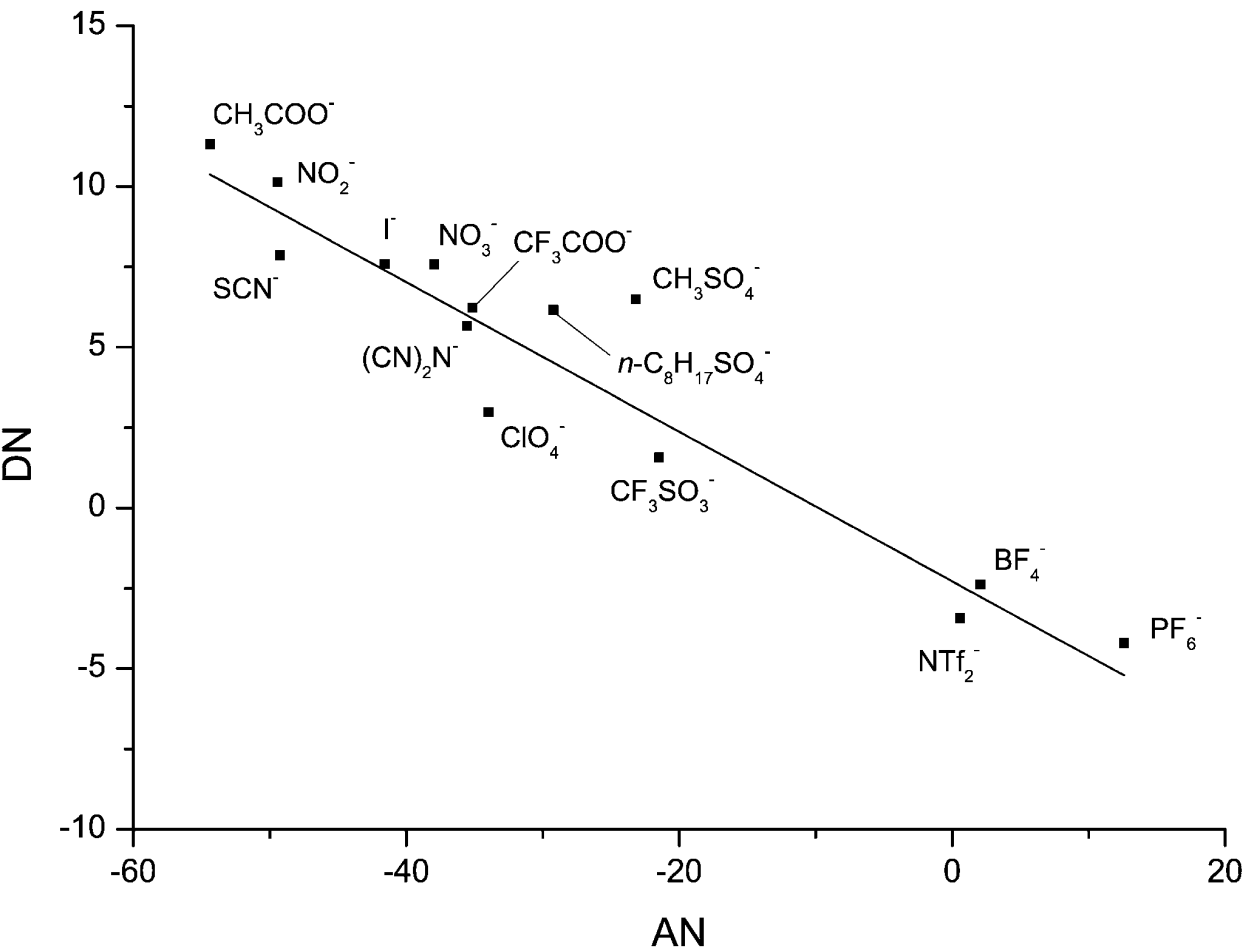
Linear relation of DN with AN for [C_4_C_1_im][X], where X is the variation of the anion; correlation *r*=0.94.

**Anion variation**: In case of ionic liquids both the anions and the cations influence the absorption spectra of the indicator, and the summary effect can be seen. Table 1 summarizes the absorption band maximum of the complexes and the obtained donor and acceptor numbers in neat 1-butyl-3-methylimidazolium (C_4_C_1_im^+^) based ionic liquids. Figure 1 shows a linear dependence of the donor and acceptor number. In conventional solvents where a solvent molecule can act as donor and/or as acceptor, for example, methanol is a strong acceptor but can also donate electrons from the lone pairs of the oxygen, and no such correlation can be found. In contrast, in ionic liquids without functional groups the cation is only acting as electron pair acceptor and the anion only as electron pair donor (additional functional groups can of course also act as acceptor and/or donor). The negative AN (DN) might imply that the cation (the anion) is actually a donor, but this seems to be a matter of referencing. In the donor–acceptor approach an arbitrarily referencing system is used. DN represents the negative Δ*H* value of the reaction of SbCl_5_ with a solvent molecule L to the adduct SbCl_5_⋅L in high dilution of DCE. Here DCE has been defined as reference solvent with DN=0 (despite the fact that it itself shows very low donor ability, which was neglected). For commonly used solvents this reaction is exothermic so that Δ*H* turns out to be positive and usual values are in the range between 0 (DN) and 100. AN on the contrary is defined as the NMR chemical shift of the ^31^P nucleus in Et_3_PO in the respective solvent (undiluted). To obtain comparable values with the DN scale this NMR shifts (measured in ppm) have been renormalized to become 100 for the (DN-important) probe SbCl_5_. Negative AN (DN) just means, that the ionic liquid in question is a weaker acceptor (donor) than nitromethane (dichloroethane). For example, in acetone (AN=12.5) Cl^−^ is as strong donor (DN=38.5); upon changing to a solvent with a higher AN the donicity of Cl^−^ shrinks (DN in methanol is only 22.6). In [C_4_C_1_im][Cl] the solvation shell consists of C_4_C_1_im^+^ cations which are accepting most of the electrons from Cl^−^ and the cation is therefore a very weak acceptor and the Cl^−^ is a weaker donor than in acetone or methanol. [BF_4_]^−^ exhibits in acetone a donor strength of 8.33 and in methanol −7.56; in C_4_C_1_im^+^ the DN is −4.18, having approximately the same donicity as [CF_3_SO_3_]^−^ in water (DN=4.0).^16^

**Table 1:** Absorption maxima of the solvatochromic indicators and calculated donor and acceptor numbers for 1-butyl-3-methylimidazolium (C_4_C_1_im^+^) based neat ionic liquids.

Cation	Anion	*λ*_max_ (Fe)^[a]^ [nm]	*λ*_max_ (Cu) [nm]	AN	DN	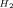 ^[a]^ [ppm]
C_4_C_1_im^+^	Cl^−^	593.1	–	–	–	10.31
C_4_C_1_im^+^	CH_3_COO^−^	590.0	702.0	−54.38	11.32	10.27
C_4_C_1_im^+^	Br^−^	590.0	–	–	–	9.94
C_4_C_1_im^+^	CF_3_COO^−^	585.1	638.0^[b]^	−35.15	6.23	9.74
C_4_C_1_im^+^	NO_2_^−^	588.9	684.0	−49.44	10.14	9.68
C_4_C_1_im^+^	I^−^	586.2	659.0	−41.58	7.59	9.68
C_4_C_1_im^+^	NO_3_^−^	586.9	645.0	−37.96	7.58	9.57
C_4_C_1_im^+^	*n*-C_8_H_17_SO_4_^−^	586.2	617.0	−29.24	6.16	9.32
C_4_C_1_im^+^	CH_3_SO_4_^−^	587.9	596.0	−23.20	6.49	9.21
C_4_C_1_im^+^	SCN^−^	585.1	690.0^[c]^	−49.26	7.86	9.15
C_4_C_1_im^+^	(CN)_2_N^−^	584.1	641.0^[c]^	−35.56	5.66	8.96
C_4_C_1_im^+^	CF_3_SO_3_^−^	580.0	601.5^[d]^	−21.47	1.57	8.91
C_4_C_1_im^+^	BF_4_^−^	578.0	540.0^[b]^	2.07	−2.38	8.64
C_4_C_1_im^+^	NTf_2_^−^	576.0	546.0	0.56	−3.44	8.56
C_4_C_1_im^+^	PF_6_^−^	577.0	516.5^[e]^	12.59	−4.21	8.38

[a] Values from reference 19. [b] Values from reference 20. [c] Values from reference 21. [d] Values from reference 22. [e] Values from reference 23.

**Figure 2:**
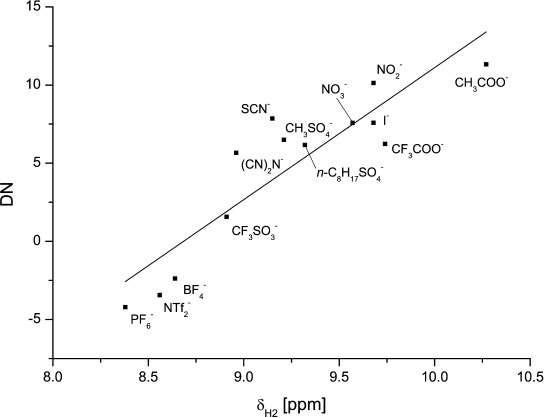
Linear correlation of DN with 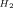 for [C_4_C_1_im][X], where X is the variation of the anion.

**Correlation between DN and NMR shifts**: It has been shown that the Kamlet–Abboud–Taft solvent parameter β (i.e., a measure of the hydrogen bond accepting (HBA) ability) is linearly correlated to the H-2 hydrogen NMR chemical shift 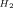 of 1-butyl-3-methylimidazolium C_4_C_1_im^+^ based ionic liquids.^19^, ^24^ In our consideration the DN value should also be correlated linearly. Figure 2 shows such a behavior. The electronic environment of the “theoretically isolated” cation C_4_C_1_im^+^ is fixed. Any alteration on the electron density is therefore dependent on the nature of the anion.

As there is no additional NMR sensitive probe molecule in the ionic liquid (the shift of a NMR probe such as triethylphosphine oxide should be dependent on donor and acceptor properties as do the solvatochromic indicators) the donor properties of the anion can be screened solely by the shift of H-2, that is, the H-2 proton acts as “built-in” indicator.^24^ From a linear correlation of 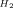 with DN and a linear correlation of AN with DN (Table 2), it is possible to determine donor and acceptor numbers for (in this case) C_4_C_1_im^+^ based ionic liquids where a solvatochromic approach is not feasible (e.g., high melting, colored or hydrolysis sensitive ionic liquids):

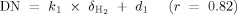
11

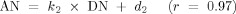
12

**Table 2:** Calculated donor and acceptor numbers for 1-butyl-3-methylimidazolium based neat ionic liquids based on the correlation of 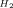 with DN and AN with DN.

Cation	Anion	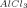	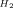 ^[a]^ [ppm]	AN	DN
C_4_C_1_im^+^	SnCl_3_^−^		9.24	−28.29	4.52
C_4_C_1_im^+^	BBr_4_^−^		9.04	−21.34	2.83
C_4_C_1_im^+^	TiCl_5_^−^		8.99	−19.61	2.41
C_4_C_1_im^+^	SbCl_6_^−^		8.68	−8.83	−0.22
C_4_C_1_im^+^	I_3_^−^		8.66	−8.14	−0.39
C_4_C_1_im^+^	SnCl_5_^−^		8.59	−5.71	−0.98
C_4_C_1_im^+^	I_5_^−^		8.52	−3.27	−1.57
C_4_C_1_im^+^	WCl_7_^−^		8.50	−2.58	−1.74
C_4_C_1_im^+^	MoCl_6_^−^		8.06	12.71	−5.47
C_4_C_1_im^+^	AlCl_4_^−^	0.17	10.29	−64.78	13.41
C_4_C_1_im^+^	AlCl_4_^−^	0.29	10.05	−56.44	11.38
C_4_C_1_im^+^	AlCl_4_^−^	0.38	9.62	−41.50	7.74
C_4_C_1_im^+^	AlCl_4_^−^	0.44	9.06	−22.04	3.00
C_4_C_1_im^+^	AlCl_4_^−^	0.5	8.39	1.24	−2.67
C_4_C_1_im^+^	Al_2_Cl_7_^−^	0.55	8.37	1.94	−2.84
C_4_C_1_im^+^	Al_2_Cl_7_^−^	0.58	8.34	2.98	−3.10
C_4_C_1_im^+^	Al_2_Cl_7_^−^	0.62	8.30	4.37	−3.44
C_4_C_1_im^+^	Al_2_Cl_7_^−^	0.64	8.27	5.41	−3.69
C_4_C_1_im^+^	Al_2_Cl_7_^−^	0.67	8.23	6.80	−4.03

[a] Values from reference 24.

with *k*_1_=8.4667, *d*_1_=−73.7101, *k*_2_=−4.1043 and *d*_2_=−9.7339. Table 2 summarizes the results calculated from the NMR shift. These are in agreement with the results for the anion variation of C_4_C_1_im^+^ ionic liquids determined by solvatochromic indicators. In the series of chloroaluminate anions it can be seen, that an ionic liquid with a low mole fraction of added AlCl_3_ exhibits strong donating abilities, whereas the donor properties are reduced as the mole fraction rises (i.e., the “basicity” of the anion decreases by increasing the amount of the Lewis acid AlCl_3_).

**Figure 3:**
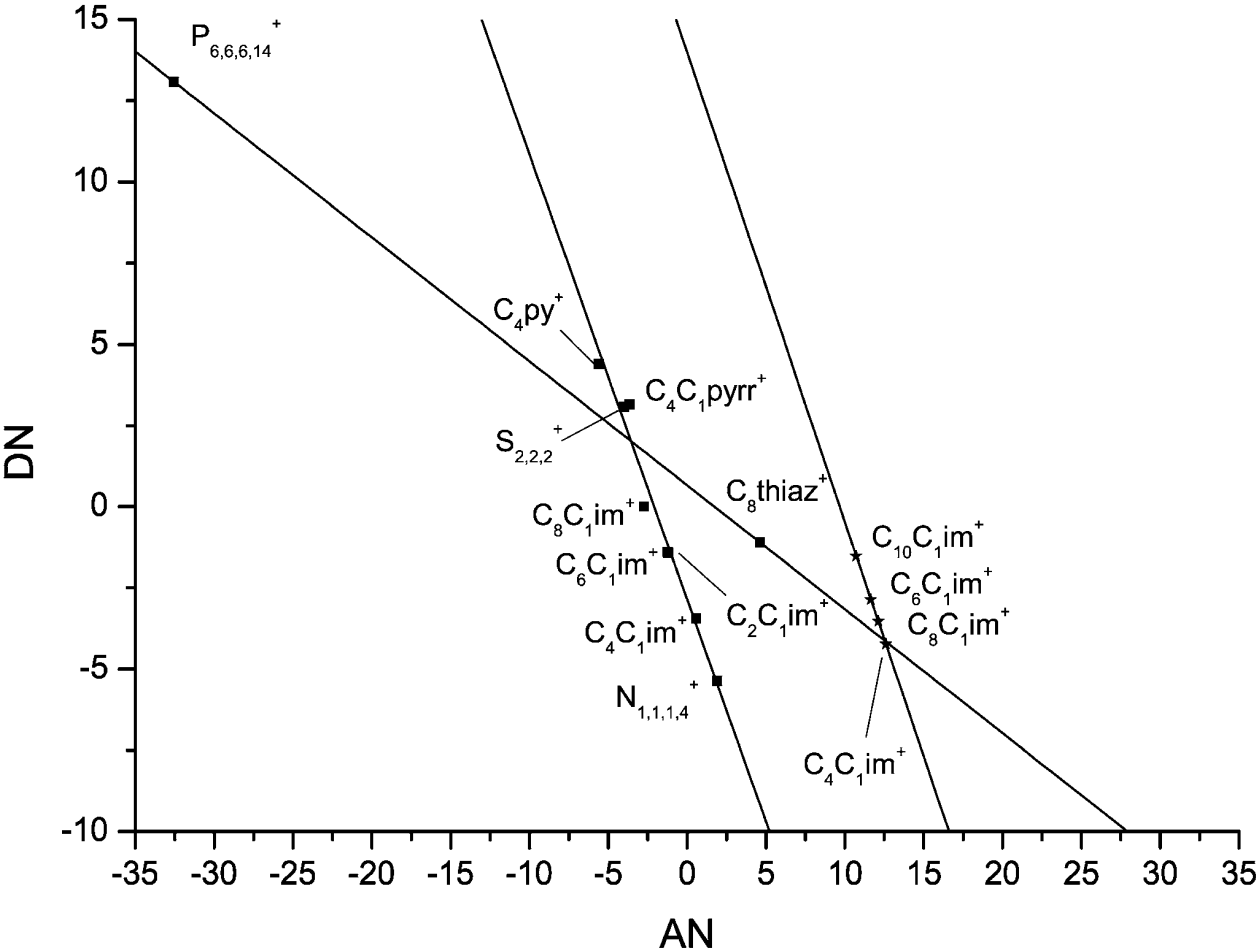
Linear correlation of DN with AN for [C][NTf_2_] (squares) and [C][PF_6_] (stars), where C is the variation of the cation.

**Cation variation**: Figure 3 shows for the variation of the cation two different correlations. Less steric demanding cations (N_1,1,1,4_^+^, S_2,2,24_^+^, C_4_py^+^, C_1_C_4_pyrr^+^, C_2_C_1_im^+^, C_4_C_1_im^+^, C_6_C_1_im^+^ and C_8_C_1_im^+^) give a steeper slope than cations with a high steric demand (P_6,6,6,14_^+^, C_8_thiaz^+^). Although choline is not sterically demanding it does not correlate with the other less sterically demanding cations. The reason for this could be the functional group OH which gives an additional possibility of interaction (e.g., hydrogen bonding, interaction of the free electron pairs of the oxygen) with a given anion. Table 3 summarizes the absorption maxima of the solvatochromic indicators and calculated donor and acceptor numbers for bis(trifluoromethylsulfonyl)imide based neat ionic liquids. The current state of knowledge from the available data allows the conclusion that the influence of the cation is driven by steric hindrance and the nature (and the number of) additional functional groups.

**Table 3:** Absorption maxima of the solvatochromic indicators and calculated donor and acceptor numbers for bis(trifluoromethylsulfonyl)imide based neat ionic liquids.

Cation	Abbr.	Anion [nm]	*λ*_max_ (Fe) [nm]	*λ*_max_ (Cu)	AN	DN
butyltrimethylammonium	N_1,1,1,4_^+^	NTf_2_^−^	573.2	546.0^[a]^	1.89	−5.35
triethylsulfonium	S_2,2,2_^+^	NTf_2_^−^	585.9	591.3	−4.00	3.09
butylpyridinium	C_4_py^+^	NTf_2_^−^	587.8	548.0^[a]^	−5.60	4.41
1-butyl-1-methylpyrrolidinium	C_1_C_4_pyrr^+^	NTf_2_^−^	586.1	545.0^[b]^	−3.64	3.17
3-octylthiazolium	C_8_thiaz^+^	NTf_2_^−^	580.6	573.7	4.60	−1.10
trihexyltetradecylphosphonium	P_6,6,6,14_^+^	NTf_2_^−^	597.3	669.2	−32.55	13.08
choline	chol^+^	NTf_2_^−^	553.0	535.8	32.61	−21.98

[a] Values from reference 20. [b] Values from reference 11.

**Figure 4:**
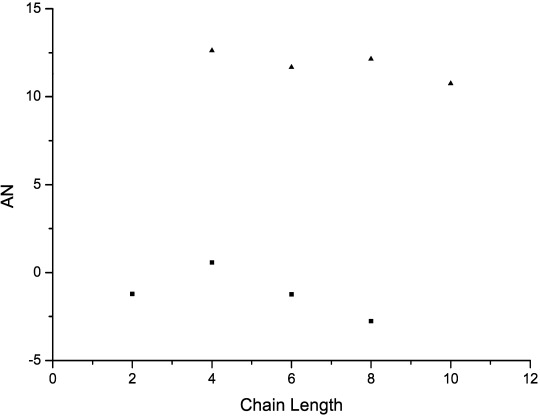
Dependence of the side chain length in [C_*n*_C_1_im][NTf_2_] and [C_*n*_C_1_im][PF_6_] on AN.

**Side chain length variation**: The interpretation of the influence of the side chain length is not straightforward. Considering a virtual, from anions unaffected C_*n*_mim cation, a linear decrease of AN would be expected due to the increasing inductive effect of a longer side chain. From the available data the results show local minima and maxima for DN and AN, respectively, in dependence of the chain length. The DN and AN values are still linearly correlated but not in the order as it would be expected from the increasing inductive effect (see Figure 4). Despite the different anion size and donor strength from PF_6_^−^ and NTf_2_^−^ a parallel displacement of the linear correlation can be observed. Influences from steric hindrance of the side chain or the size of the anion (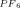 ≥ 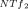) and an ordering effect from side chain stacking must be taken into account. Table 4 lists the absorption maxima of the solvatochromic indicators and calculated donor and acceptor numbers for imidazolium based neat ionic liquids with fixed anion. More data for different anions and especially for the structure of ionic liquids in the liquid state is needed for a correct interpretation.

**Table 4:** Absorption maxima of the solvatochromic indicators and calculated donor and acceptor numbers for imidazolium based neat ionic liquids with fixed anion.

Cation	Anion	*λ*_max_ (Fe) [nm]	*λ*_max_ (Cu) [nm]	AN	DN
C_4_C_1_im^+^	PF_6_^−^	577.0	516.5	12.61	−4.23
C_6_C_1_im^+^	PF_6_^−^	579.0	516.5	11.66	−2.87
C_8_C_1_im^+^	PF_6_^−^	578.0	516.5	12.13	−3.54
C_10_C_1_im^+^	PF_6_^−^	581.1	516.5	10.74	−1.53
C_2_C_1_im^+^	NTf_2_^−^	579.0	547.0	−1.21	−1.41
C_4_C_1_im^+^	NTf_2_^−^	576.0	546.0	0.58	−3.46
C_6_C_1_im^+^	NTf_2_^−^	579.0	547.0	−1.23	−1.38
C_8_C_1_im^+^	NTf_2_^−^	581.1	548.5	−2.75	0.03

Acceptor numbers determined from Kamlet–Abboud–Taft parameters,^19^ Raman^25^ and ^31^P NMR spectroscopy^26^, ^27^ were already reported for ionic liquids. Table 5 compiles some values for AN from literature. The apparent donor and acceptor numbers established by our approach do not resemble the previously reported values for ionic liquids. The main difference lies in the mutual influence of cations and anions, which are not taken into account by the other authors. Calculating acceptor numbers without considering the influence of the counter ion [using Eq. (1)] AN’s comparable to values determined by other methods are obtained. Polarity scales like Kamlet–Abboud–Taft and Reichardt’s *E*_T_ do not consider this ionic interaction. The same is true for the calculation of AN for ionic liquids by ^31^P NMR. Any probe molecule dissolved in an ionic liquid is dependent on the donor and acceptor properties of the ionic liquid. Therefore the chemical shift (*δ*_P_) of the probe triethylphosphine oxide is dependent on the solvent used. Using conventional solvents the donor property of a solvent is negligible, but not in ionic liquids, were the solvation shell of the probe molecule coordinated to the cation (Lewis acid) consists of anions (Lewis base). Therefore all previously reported acceptor numbers are too high since the lowering effect of the anion is not considered. To visualize the importance of mutual interactions to acid–base considerations, one might compare AN and DN (representing Lewis acidities and basicities) with “simple” Brønsted relations. Taking H_3_BO_3_ for example, which is a very weak acid an aqueous solutions (i.e., its grade of deprotonation is very low) becomes strongly deprotonated in liquid ammonia and is acting in reactions accordingly in such media. On the other hand, strong bases will lose their basicity in liquid ammonia. The situation obviously becomes reversed using acidic media such as water-free acetic acid as solvents.

**Table 5:** Comparison of AN values determined by different approaches.

Cation	Anion	AN ref. 5	AN ref. 16	AN calcd Eq. (1)	AN ref. 6	AN calcd Eq. (9)
C_4_C_1_im^+^	NTf_2_^−^		30.05	25.99	25.20	2.37
C_4_C_1_im^+^	BF_4_^−^		29.26	28.77	26.90	4.55
C_4_C_1_im^+^	PF_6_^−^		29.66	29.18	27.70	12.37
C_4_C_1_im^+^	Cl^−^		23.41	–	26.9	–
C_8_C_1_im^+^	Cl-AlCl_3_^−^	91.81				
C_8_C_1_im^+^	Cl-GaCl_3_^−^	45.85				
C_8_C_1_im^+^	Cl-InCl_3_^−^	57.11				

These results indicate that ionic liquid cations and anions cannot be discussed separately; the mutual influence of the counter ion has to be considered; this means that the acceptor (donor) properties are not only a function of the cation (anion), as it is usually suggested for the Kamlet–Abboud–Taft parameters *α* and *β*, but also a function on the anion (cation). Increasing the basicity, for example, from [BF_4_]^−^ to [Cl]^−^ the accepting ability is reduced. Increasing the acidity, for example, from [S_2,2,2_]^+^ to [chol]^+^ the donating ability of [NTf_2_]^−^is reduced.

## Conclusion

Frequently the polarity of ionic liquids is described to behave like conventional solvents^28^ (depending on the ion combination) for example, C_4_C_1_im^+^ based ionic liquids are usually compared to lower alcohols.^11^ Here we have to disagree with the literature, as our results clearly show that ionic liquids cannot be considered to behave like conventional solvents, they have to be regarded as a unique class of solvents. DN and AN of cations and anions are influenced by the solvent.^16^, ^17^ Based on this fact and considering ionic liquids as cations (anions) solvated by anions (cations) a simple concept of mutual interactions was developed. For solvent describing scales this has not been taken into account (as for most reactions this can be neglected), however, it leads to the fact that basicity and acidity related scales are usually not in a simple relationship. It can be shown, that the data on the Eyring activation parameters^9^ are in principal related to DN of the anions in conventional solvent^16^ showing that the Gutmann AN–DN approach is consistent. Unfortunately, no DN for anions in ionic liquids are available up to date to further verify the proposed concept.

## Methods

The ionic liquids used in the study were purchased from Sigma–Aldrich (Steinheim, Germany) and TCI (Tokyo, Japan) or were a donation from AC^2^T (Wiener Neustadt, Austria) and the Institute of Applied Synthetic Chemistry (Vienna University of Technology, Austria) or were prepared according to literature protocol.^24^ Ionic liquids can absorb considerable amounts of water from air. Therefore the ionic liquids were dried at 40 °C at reduced pressure for at least two days. The dried ionic liquids were stored at room temperature in a desiccator to the exclusion of light. A small amount of the indicator ([Cu(acac)(tmen)][ClO_4_] or [Fe(phen)_2_(CN)_2_]) dissolved in dichloroethane (DCE) is added to 400 μL ionic liquid dissolved in DCE. This ensures a homogeneous distribution of the indicators. The solvent is then removed at reduced pressure (approx. 10^−3^ mbar). The so prepared samples are transferred into a cuvette (108.002-QS, Hellma GmbH, Mülheim, Germany) and are measured against the pure, dried ionic liquid. UV-Vis spectra were obtained on a Perkin–Elmer Lambda 900 (Shelton, CT, USA) or a Carl Zeiss MCS 400 (Jena, Germany) spectrophotometer in transmission mode at room temperature. The spectral range was 300 nm to 1000 nm.
